# The Floor Is Lava: Halving Natural Genomes with Viaducts, Piers, and Pontoons

**DOI:** 10.1089/cmb.2023.0330

**Published:** 2024-04-22

**Authors:** Leonard Bohnenkämper

**Affiliations:** Faculty of Technology, Center for Biotechnology (CeBiTec), Bielefeld University, Bielefeld, Germany.

**Keywords:** DCJ-Indel, genome halving, genome rearrangement, integer linear programming, natural genomes

## Abstract

Whole Genome Duplications (WGDs) are events that double the content and structure of a genome. In some organisms, multiple WGD events have been observed while loss of genetic material is a typical occurrence following a WGD event. The requirement of classic rearrangement models that every genetic marker has to occur exactly two times in a given problem instance, therefore, poses a serious restriction in this context. The Double-Cut and Join (DCJ) model is a simple and powerful model for the analysis of large structural rearrangements. After being extended to the DCJ-Indel model, capable of handling gains and losses of genetic material, research has shifted in recent years toward enabling it to handle natural genomes, for which no assumption about the distribution of markers has to be made. The traditional theoretical framework for studying WGD events is the Genome Halving Problem (GHP). While the GHP is solved for the DCJ model for genomes without losses, there are currently no exact algorithms utilizing the DCJ-Indel model that are able to handle natural genomes. In this work, we present a general view on the DCJ-Indel model that we apply to derive an exact polynomial time and space solution for the GHP on genomes with at most two genes per family before generalizing the problem to an integer linear program solution for natural genomes.

## 1. INTRODUCTION

Whole Genome Duplications (WGDs) are evolutionarily far-reaching events in which the content and structure of a genome is doubled. There are lineages with multiple known WGD events (Dehal and Boore, 2005) and WGDs are typically followed by large-scale loss of genetic content (Scannell et al., 2006).

For genome rearrangement studies, the classic framework for analyzing WGD events is the Genome Halving Problem (GHP). Broadly speaking, the task formulated by the GHP is, given a present-day genome, to reconstruct a genome with the specific structure that is expected to arise after a WGD. This genome is required to have the minimal distance under some rearrangement model to the given present-day genome. A popular rearrangement model is the Double-Cut and Join (DCJ) model (Yancopoulos et al., 2005; Bergeron et al., 2006). This model has been applied to the GHP by Mixtacki (2008), but as a pure rearrangement model, this solution was not able to account for the losses frequently observed after WGD events. Savard et al. (2011) were able to provide a solution to the GHP under DCJ that accounts for lost markers, but not for the loss event itself, meaning they were not able to account for its contribution to the distance. This is likely due to the fact that the DCJ-Indel model, which accounts for costs of segmental Indel events, was only first formulated around the same time (Braga et al., 2011).

Another issue is the limitation of GHP solutions on input genomes with markers occurring at most two times. So far, there are multiple publications addressing this problem in a distance context (Shao et al., 2015; Bohnenkämper et al., 2021), but with many examples of multiple rounds of WGDs and other duplication events occurring in tandem, we can expect markers to occur more than two times in many practical GHP scenarios. We call these markers with more than two occurrences *ambiguous*, and genomes with arbitrary distributions of markers *natural genomes*.

The need for theoretical inclusion of natural genomes in the context of WGDs has been recognized by Avdeyev and Alekseyev (2020), who created an approximate solution for a generalized variant of the GHP. However, as far as we are aware, no exact solutions for the GHP under the DCJ-Indel model have been proposed as of yet.

In Sections 2 and 3, we develop a simple view on the DCJ-Indel model that can be derived independently from the ones presented in Braga et al. (2011) and Compeau (2013). We apply this conceptualization to solve the GHP for genomes without ambiguous, but lost or gained markers in polynomial time in Section 4. We note that the GHP is NP-hard for natural genomes under the DCJ-Indel model. Therefore, we give an integer linear program (ILP) to solve the generalized problem for natural genomes in Section 5. We evaluate the ILPs performance in Section 6 on both simulated and real data.

## 2. PROBLEM DEFINITION

For the purposes of this work, we view a genome G as a graph $\left(X_{G},M_{G}\cup A_{G}\right)$. G consists of markers $m:=\left\{m^{t},m^{h}\right\}\in M_{G}$ and their *extremities*, that is their beginning *m^t^* (read “*m* tail”) and an end *m^h^* (read “*m* head”), which make up the genome's vertices, $X_{G}:=\left\{m^{t}|m\in M_{G}\right\}\cup \left\{m^{h}|m\in M_{G}\right\}$. The structure of a genome is captured by undirected edges $A_{G}$, called *adjacencies*. As a shorthand notation, we write *ab* for an adjacency {a,b}. Both the set of adjacencies $A_{G}$ and the set of markers $M_{G}$ form a matching on the extremities $X_{G}$.

**FIG. 1.**
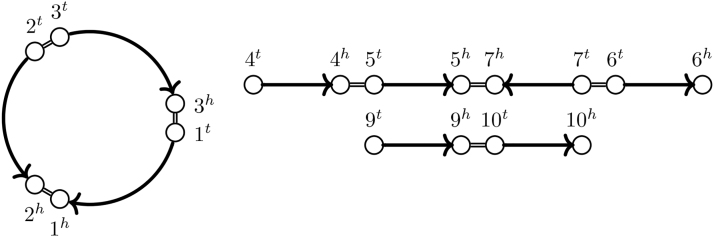
Genome with one circular and two linear chromosomes. Adjacencies are drawn as double lines.

Each path in G is simple and alternates between markers and adjacencies. We call a path $p=p_{1},p_{2},\ldots ,p_{l-1},p_{l}$ a *chromosome segment* if it starts and ends with a marker, that is, $\left\{p_{1},p_{2}\right\},\left\{p_{l-1},p_{l}\right\}\in M_{G}$. We call p a *chromosome* if there are either no adjacency edges incident to *p*_1_ or *p_l_* or $p_{l}p_{1}\in A_{G}$. If $p_{l}p_{1}\in A_{G}$, we refer to *p* as *circular*. Otherwise (i.e., $\deg\left(p_{1}\right)=\deg\left(p_{l}\right)=1$), we refer to it as *linear* and call *p*_1_ and *p_l_ telomeres*. An example of a genome is given in Figure 1.

Each marker is unique, so to find structural similarities, we borrow a concept from biology, namely *homology*. We model homology as an equivalence relation on the markers, that is, m≡n for some $m,n\in M_{G}$. We also derive an equivalence relation on extremities with $m^{t}\equiv n^{t}\wedge m^{h}\equiv n^{h}\Leftrightarrow m\equiv n$, but $m^{t}n^{h}\forall m,n$, and a derived equivalence relation on adjacencies, ab≡cd⇔a≡c∧b≡d or a≡d∧b≡c. The equivalence class [m] of a marker *m* is also called a *family*. We call a marker *m singular* if it has no homolog, but itself in the given problem instance. We call a circular (linear) chromosome consisting only of singular markers a *circular (linear) singleton*.

We now introduce the Supernatural Graph (SNG), a graph structure that will be useful later and meanwhile illustrates our conceptualization of homology.

**Definition 1.** *The* SNG(G,≡) *of a genome* G *with homology* (≡) *is a graph with vertices* $V=X_{G}$ *and two types of undirected edges* $E=E_{\gamma }\cup E_{\xi }$, *namely* adjacency edges $E_{\gamma }:=A_{G}$ *and* extremity edges $E_{\xi }=\left\{\left\{g,h\right\}|g,h\in X_{G},g\neq h,g\equiv h\right\}$.

**FIG. 2.**
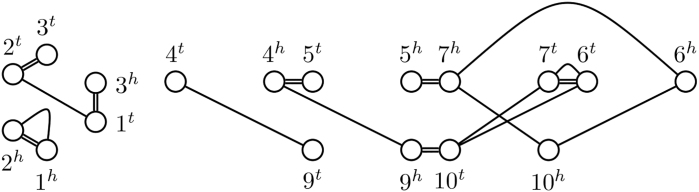
SNG for genome A from Figure 1 resulting from a homology relation $\left(\equiv_{a}\right)$ with the following families: {1,2},{3},{4,9},{5},{6,7,10}. Extremities are arranged in the same way as in Figure 1.

This graph is a variant of the *natural graph* as used in Mixtacki (2008) with the difference that adjacencies are modeled as edges instead of vertices and the possibility of more than one homolog per extremity. An example of an SNG can be found in Figure 2.

Genomes following a WGD possess a very particular structure due to the nature of this event. Not only is its marker content duplicated, but also its adjacency information is preserved. On some homologies this is easy to detect, namely if each family contains at most two markers. We call such homologies *resolved*. For more complex homologies, it is helpful to find an artificial resolved homology, called a *matching*.

**Definition 2.** *A* matching $\left(\equiv^{⋆}\right)$ *on a given homology* (≡) *is a resolved homology for which holds* $m\equiv^{⋆}n\Rightarrow m\equiv n$ *for any pair of markers* m,n.

**FIG. 3.**
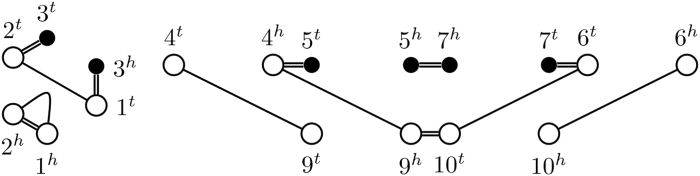
The simple SNG for genome A from Figure 1 resulting from a resolved homology relation $\left({\equiv^{⋆}}_{a}\right)$ is a consistent decomposition of the SNG of Figure 2 because $\left({\equiv^{⋆}}_{a}\right)$ is a matching on $\left(\equiv_{a}\right)$. $\left({\equiv^{⋆}}_{a}\right)$ has the following families: {1,2},{3},{4,9},{5},{6,10},{7}. Lava vertices (Section 3) filled black.

We note that when the homology is resolved, at most one extremity edge connects to each vertex in the SNG. Because adjacencies form a matching on the extremities, the resulting SNG consists of only simple cycles and paths. We, therefore, call such an SNG *simple*. An example is given in Figure 3. We also note that the simple SNG $SNG\left(G,\equiv^{⋆}\right)$ of a genome G with matching $\left(\equiv^{⋆}\right)$ on the original homology (≡) contains all adjacency edges of SNG(G,≡) and a subset of its extremity edges. The simple SNG $SNG\left(G,\equiv^{⋆}\right)$ is called a *consistent decomposition* of SNG(G,≡).

A genome resulting from a WGD is called structurally doubled (SD). To formally define that each marker as well as adjacency information is doubled, we define it in terms of a matching.

**Definition 3.** *A genome* G *is called* SD *under homology* (≡), *if and only if there is a matching on* (≡)*, under which each marker and adjacency is in an equivalence class with exactly one other element of* G.

**FIG. 4.**
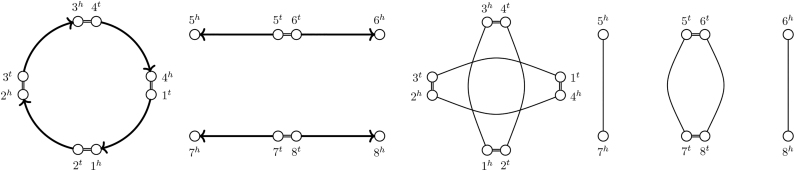
The genome ℬ depicted on the left is SD under the resolved homology relation $\left({\equiv^{⋆}}_{b}\right)$ with families {1,3},{2,4},{5,7},{6,8}, as illustrated by the SNG on the right. Note that there are also unresolved homology relations, for example, $\left(\equiv_{b}\right)$ with families {1,3},{2,4,6,8},{5,7}, for which ℬ is SD because $\left({\equiv^{⋆}}_{b}\right)$ is a matching on $\left(\equiv_{b}\right)$.

Note that for a resolved homology, this definition corresponds directly with the definition of a *perfectly duplicated* genome as in Mixtacki (2008). We give an example of an SD genome in Figure 4.

Over time this specific structure after a WGD is typically erased by rearrangements and losses. Rearrangements in our transformation distance are modeled by the Double-Cut and Join (DCJ) operation. In principle, a DCJ operation cuts in two places (adjacencies or telomeres) in the genome and reconnects the incident extremities. More formally, we can write as in Bergeron et al. (2006):

**Definition 4.** *A* DCJ operation *transforms up to two adjacencies* $ab,cd\in A_{A}$ *or telomeres* s,t *of genome* A *in one of the following ways:*

- ab,cd→ac,bd *or* ab,cd→ad,bc • ab→a,b
- ab,s→as,b *or* ab,s→bs,a • s,t→st

To account for losses or possibly gains following a WGD, we introduce segmental insertions and deletions.

**Definition 5.** *An* insertion *of length l transforms a genome* A *into* A′ *by adding a chromosome segment* $p=p_{1},p_{2},\ldots ,p_{2l-1},p_{2l}$ *to the genome. It may additionally have either* $p_{2l}p_{1}\in A_{A\prime }$*, apply the transformation* $ab\to ap_{1},p_{2l}b$ *for an adjacency ab or the transformation* $s\to p_{1}s$ *for a telomere s*.

*A* deletion *of length l removes the chromosome segment* $p=p_{1},\ldots ,p_{2l}$ *and creates the adjacency ab if previously* $ap_{1},p_{2l}b\in A_{A}$.

To reconstruct an SD ancestor to a present-day genome, we require this ancestor to be as closely related as possible under our model. We define the GHP first as an abstract problem with an arbitrary set of operations.

**Problem 1** (GHP). *Given a genome* G *and a homology* (≡)*, find the minimum length* h(G,≡)=l *for any legal scenario* $G=G_{0}\to^{o_{1}}G_{1}\ldots ,\to^{o_{l}}G_{l}$ *transforming* G *into a SD genome* $G_{l}$ *with operations* 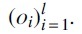 *We call* h(G,≡) *the* halving distance *of* G *under* (≡).

For the DCJ-Indel model, the operations used are DCJs (Definition 4) and Indels (Definition 5) of arbitrary length. Initially, any sequence of DCJs and Indels is legal. However, we will soon see that it makes sense to restrict legal scenarios to avoid an excess of Indels.

**Problem 2** (DCJ-Indel GHP). *Given a genome* G *and a homology* (≡)*, find the minimum length* $h_{DCJ}^{id}\left(G,\equiv \right)=l$ *for any scenario* $G=G_{0}\to^{o_{1}}G_{1}\ldots ,\to^{o_{l}}G_{l}$ *transforming* G *into a SD genome* $G_{l}$ *with DCJ and Indel operations* 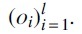 *We call* $h_{DCJ}^{id}\left(G,\equiv \right)$ *the DCJ-Indel halving distance of* G *under* (≡).

The original DCJ-Indel model allowed Indel operations only for singular markers, in that only singular markers could be deleted and at most one homolog of a singular marker could be inserted (Braga et al., 2011). When restricting legal scenarios in Problem 2 to only include such Indel operations, we speak of the *restricted halving distance*. For a resolved homology $\left(\equiv^{⋆}\right)$, we write the restricted halving distance as $\bar{h_{DCJ}^{id}}\left(G,\equiv^{⋆}\right)$.

Note that the restricted halving distance only works properly for a resolved homology, because in an unresolved homology, ambiguous families with odd numbers of markers cannot be dealt with. However, we can use a matching to circumvent this problem. Since being SD implies a matching $\left(\equiv^{⋆}\right)$ for the reconstructed ancestor $G_{l}$, by applying the rearrangement scenario backward, we know that there must exist the same matching on G, although some markers might not have a homolog. The resulting problem formulation can be used to solve Problem 2 as shown in Bohnenkämper (2023b, section A.1).

**Problem 3.** *Given a genome and a homology* (≡)*, find a matching* $\left(\equiv^{⋆}\right)$ *on* (≡)*, such that the* restricted DCJ-Indel halving distance $\bar{h_{DCJ}^{id}}\left(G,\equiv^{⋆}\right)$ *is minimized.*

A typical issue that arises in these types of problems once one introduces segmental insertion and deletion operations is that without restrictions, the shortest way to sort is often by just deleting one genome and inserting another, resulting in an empty matching. We, therefore, work within an established framework, namely *Maximum Matching* (Fertin et al., 2009).

**Definition 6.** *A matching* $\left(\equiv^{+}\right)$ *on a homology* (≡) *is called a* maximum matching *if for each family under* (≡) *there is at most one singular marker under* $\left(\equiv^{+}\right)$.

We can then formulate the GHP for the Maximum Matching model as follows:

**Problem 4.** *Given a genome and a homology* (≡)*, find a maximum matching* $\left(\equiv^{+}\right)$ *on* (≡)*, such that the restricted DCJ-Indel halving distance* $\bar{h_{DCJ}^{id}}\left(G,\equiv^{+}\right)$ *is minimized.*

For resolved homologies $\left(\equiv^{⋆}\right)$, we note that a maximum matching must have the same families, that is $\left(\equiv^{⋆}\right)=\left(\equiv^{+}\right)$. Thus, for resolved homologies, we do not need to determine a matching. For arbitrary homologies, we find the following:

**Proposition 1.** *Determining a maximum matching* $\left(\equiv^{+}\right)$ *on a natural genome* G *with homology* (≡)*, such that its restricted DCJ-Indel halving distance* $\bar{h_{DCJ}^{id}}\left(G^{\equiv^{+}}\right)$ *is minimized, is an NP-hard problem.*

A proof for this proposition based on Caprara's alternating cycle decomposition problem (Caprara, 1997) is given in Bohnenkämper (2023b, section D).

This proof, but also our derivation of the halving distance formula, relies heavily on properties of the SNG. These properties are shared with other data structures, such as the Multi-Relational Diagram in Bohnenkämper et al. (2021). To make our results as reusable as possible, we start by making observations about a generalized version of the SNG:

**Definition 7.** *A* rearrangement graph $G=\left(V,E_{\gamma }\cup E_{\xi },g\right)$ *is an undirected graph with two types of edges, namely a matching* $E_{\gamma }$ *called* adjacency edges, *additional edges* $E_{\xi }$ *called* extremity edges *and a labeling g assigning each vertex* v∈V *to a genome* g(v) *with* g(v)=g(u) *if* $uv\in E_{\gamma }$.

Similar to the SNG, we refer to rearrangement graphs with at most one extremity edge connecting to each vertex as *simple*. Because then $E_{\gamma }$ and $E_{\xi }$ both form matchings on *V*, simple rearrangement graphs too consist only of simple cycles and simple paths. We make a few general observations about the DCJ-Indel model on simple rearrangement graphs in Section 3 before coming back to the concrete case of the halving problem in Section 4.

## 3. THE PROPERTIES OF SIMPLE REARRANGEMENT GRAPHS

In this section, we examine the general properties of rearrangement graphs, which we will later use in Section 4 to derive our result on the halving distance. We denote the universe of simple cycles and paths by Ω and the components of a concrete simple rearrangement graph G by Ω(G). The universe here means the collection of all possible such components in all possible rearrangement graphs. Oftentimes, we are interested in certain subsets of Ω(G), such as the set of cycles or paths and the way they react if a DCJ operation is applied to the adjacencies and telomeres of the graph. To that end, we refer to any subset K⊂Ω as a *component subset* and define K(G):=K∩Ω(G). We refer to K(G) as an *instance of a component subset.*

We will characterize DCJ and Indel operations by their effects on different quantities, such as cardinalities of component subset instances. To that end, we define the following:

**Definition 8.** *Given a rearrangement graph* G *before and a rearrangement graph* G′ *after a given sequence of operations o as well as a quantity* q(G) *before and the same quantity* q(G′) *after the operation, we define the* difference in *q* induced by *o as*

(1) $$\Delta_{o}q=q\left(G\prime \right)-q\left(G\right).$$

Before we can characterize the effect of different operations, we need to distinguish between different component subsets by different characteristics, such as the genome of certain vertices or the number of extremity edges. We use a similar distinction as in Compeau (2013). We call a component *odd* if the number of extremity edges it contains is odd and *even* otherwise. We denote the component subset of all cycles by C and those of even and odd cycles by $C_{\circ }$ and $C_{|}$, respectively. For paths, we opt for a more fine-grained distinction. We refer to the vertices a path starts or ends in as *endpoints*. Since endpoints have degree 1, they either lack an extremity edge or an adjacency edge. We refer to vertices without an extremity edge as *lava vertices* and denote them by their genome in lower-case letters (e.g., *a*). We refer to the rest of the vertices as *safe vertices* and call those vertices without an incident adjacency edge, *telomeres*. We denote telomeres by their genome in upper-case letters (e.g., *A*). Note that a vertex can both be a lava vertex and a telomere. We can then write the component subset of all paths with endpoints α,β as $P_{\alpha \beta }$.

**FIG. 5.**
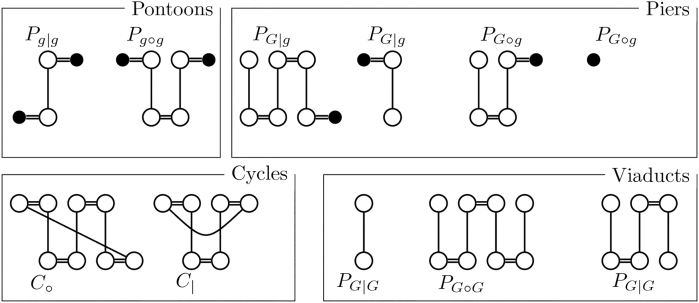
Classification of components in a simple SNG. Lava vertices filled black.

We denote the subset of even paths of this kind by $P_{\alpha \circ \beta }$ and for odd paths as $P_{\alpha |\beta }$. For example, the component subset of odd paths ending in a telomere in a genome A and a lava vertex in a genome ℬ is denoted by $P_{A|b}$. As a more intuitive classification, we refer to paths ending in two distinct lava vertices as *pontoons*, to paths ending in two safe telomeres as *viaducts* and to paths ending in both a telomere and a lava vertex as *piers*. As a shorthand, we use the notation k:=|K(G)| for the cardinality of an instance of a component subset K and *K* for a generic member K∈K(G). An example of this notation as well as of the terms we introduced here is given in Figure 5.

To not create chimeric genomes, we only allow operations if all vertices involved have the same genome label. We can then conceptualize a DCJ operation not as an operation ab,cd→ac,bd transforming adjacencies ab,cd of a rearrangement graph G into ac,bd of G′, but as transforming the components $K_{ab},K_{cd}\to K_{ac},K_{bd}$ containing these adjacencies with $K_{ab},K_{cd}\in \Omega \left(G\right),K_{ac},K_{bd}\in \Omega \left(G\prime \right)$. As an immediate result from this notation, we can see that for any component subset K, the instances K(G) and K(G′) can differ by at most four elements, that is $K\left(G\right)\setminus K\left(G\prime \right)\subseteq \left\{K_{ab},K_{cd}\right\}$ and $K\left(G\prime \right)\setminus K\left(G\right)\subseteq \left\{K_{ac},K_{bd}\right\}$. We call the components whose adjacencies are transformed *sources* and the components resulting from the operation *resultants*. All DCJ operations (see Definition 4) have at most two adjacencies as input and output and thus at most two sources and resultants, so we conclude:

**Observation 1.** *For any component subset* K *and any DCJ operation d holds*

(2) $$-2\leq \Delta_{d}|K|\leq 2.$$

We notice that this can be generalized for any number of component subsets as long as they are disjoint.

**Corollary 1.** *For any collection of disjoint component subsets L and any DCJ operation d holds*

(3) $$-2\leq \Delta_{d}\sum_{K\in L}|K|\leq 2.$$

**FIG. 6.**
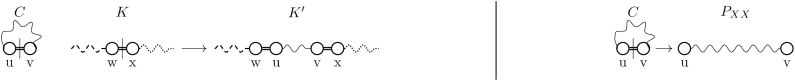
The only DCJ operations reducing the number of cycles in a rearrangement graph are of the form *C*, *K* → *K*′, or *C* → *P_XX_*. Squiggled lines represent arbitrary paths in the graph.

Cycles form a special case of Observation 1. Notice that a DCJ operation with a cycle *C* and another component *K* as sources will always integrate the cycle into *K*, forming a composite component K′. The only other way to reduce the number of cycles is to linearize it, obtaining a viaduct (see also Fig. 6).

**Observation 2.** *The only operations reducing the number of cycles are of the form* C,K→K′ *or* $C\to P_{XX}$*, with K,*K′ *some component and* $P_{XX}$ *an even or odd viaduct.*

We also notice that the cardinality of the more specific component subsets of even and odd cycles can only be reduced in the same manner. All DCJ operations have an inverse. Therefore, the only operations increasing the respective cardinalities must be the “mirror image” of those seen in Figure 6.

**Corollary 2.** *For the component subsets of even and odd cycles* $C_{\circ },C_{|}$ *and any DCJ operation d holds* $-1\leq \Delta_{d}|C_{\circ }|\leq 1$ *and* $-1\leq \Delta_{d}|C_{|}|\leq 1$.

For even cycles, things are even more specific. In this case, for $C\to P_{XX}$ the viaduct $P_{XX}$ will always be even. Additionally, for C,K→K′, the components *K* and K′ do not differ in parity or endpoints. We can thus observe:

**Observation 3.** *If the number of even cycles is changed by DCJ d (*$\Delta_{d}|C_{\circ }|\neq 0$*), the number of piers, pontoons, and odd viaducts does not change, that is,* $\Delta_{d}|P_{\alpha \left(*\right)\beta }|=0$ *for any* α,β∈{X,x,Y,y} *and* (∗)∈{∘,|} *with* α≠β *or* (∗)=|.

**FIG. 7.**
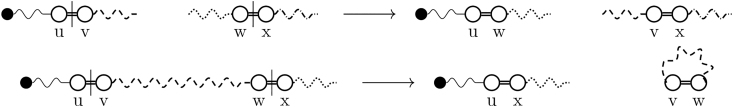
DCJ operations involving at least one pier or pontoon as a source have at least one resultant that is a pier or pontoon.

Next, we observe that DCJ operations affect only adjacency edges, so lava vertices in sources are transferred to resultants. Thus, at most one viaduct can be among the resultants of an operation with a lava vertex in its sources (see also Fig. 7).

**Observation 4.** *If the number of any type of pier or pontoon is reduced by DCJ d* 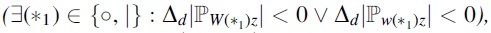 *the number of any type of viaduct can increase by at most 1, that is,* 
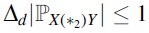
 *for any genomes* X,X,W,Z*,* $\left({*}_{2}\right)\in \left\{\circ ,|\right\}$.

**FIG. 8.**
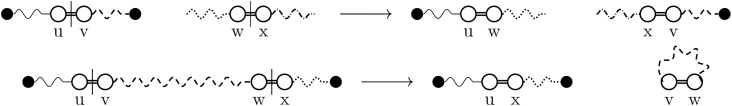
DCJ operations with a pontoon as a source either result in two components with at least one lava vertex each or a pontoon and a cycle.

Pontoons contain two lava vertices, which are distributed to both resultants (see also Fig. 8). Thus, if their number is reduced, the number of viaducts cannot increase.

**Observation 5.** *If DCJ d reduces the number of any type of pontoons* 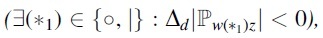 *the number of any type of viaduct does not increase, that is,* 
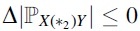
 *for any genomes* X,X,W,Z*,* $\left({*}_{2}\right)\in \left\{\circ ,|\right\}$.

Lastly, a DCJ operation cannot change the number of extremity edges in the components it affects. Thus, the total parity of sources and resultants of a DCJ operation is preserved. To capture this, we use the notation $\left({*}_{i}\right)+\left({*}_{j}\right)$ for arbitrary $\left({*}_{i}\right),\left({*}_{j}\right)\in \left\{\circ ,|\right\}$ with $\left({*}_{i}\right)+\left({*}_{j}\right)=\circ$ if $\left({*}_{i}\right)=\left({*}_{j}\right)$ and $\left({*}_{i}\right)+\left({*}_{j}\right)=|$ otherwise. We can then write:

**Observation 6.** *For any DCJ operation d of the form* 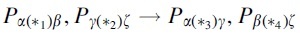 *holds* $\left({*}_{1}\right)+\left({*}_{2}\right)=\left({*}_{3}\right)+\left({*}_{4}\right)$.

**FIG. 9.**
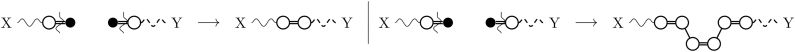
Uni-indels transform the rearrangement graph in similar ways, joining two paths ending in a lava vertex into a path or cycle (if X,Y are connected). Left: Deletion of a single marker. Right: Insertion of a single adjacency.

We have so far thoroughly investigated the effects of the DCJ operation. We now briefly touch on Indel operations. Since we have seen in Problem 3 that we can reformulate the GHP, such that only singular markers have to be part of Indels, we permit only deletions removing lava vertices or insertions creating homologs for lava vertices. First, we study only the deletion of a single marker and the insertion of a single adjacency, which refer to as *uni-Indels* collectively. While a uni-Indel deletion is always a legal operation under the DCJ-Indel model, a uni-Indel insertion is not necessarily legal. However, we will soon see that they are useful for describing insertions, so we permit them as an intermediate step of an incomplete insertion. Both types of uni-Indels remove two lava vertices from the graph and connect their adjacent vertices into one component. The similarity is visualized in Figure 9. Since the number of extremity edges is changed by 2 if at all, the total parity is again conserved.

**Observation 7.** *Uni-Indels are of the form*


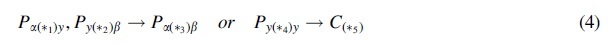


*for some genomes* X,X,Z *where* α∈{x,X},β∈{z,Z}, $\left({*}_{1}\right)+\left({*}_{2}\right)=\left({*}_{3}\right)$, *and* $\left({*}_{4}\right)=\left({*}_{5}\right)$.

We can conceptualize a deletion of *l* markers as first summarizing the stretch of markers only separated by l−1 pontoons of length 0 and then applying a uni-Indel to the “summary” marker.

**Observation 8.** *For a deletion* δ *of markers* $m_{1},\ldots ,m_{l}$ *there is a uni-Indel u on a rearrangement graph, where* $m_{1},\ldots ,m_{l}$ *are replaced by* $\tilde{m}$ *with* $\Delta_{\delta }|K|=\Delta_{u}|K|$ *for any component subset* K *that does not contain s of length* 0.

We can conceptualize an insertion of *l* markers as first inserting the circular chromosome $m_{1},\ldots ,m_{l}$ (i.e., by *l* uni-Indels) and then possibly applying a single DCJ-operation integrating the chromosome into the target adjacency.

**Observation 9.** *For an insertion* ι *of markers* $m_{1},\ldots ,m_{l}$ *there are uni-Indels* 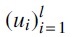 *and a DCJ operation d on the same rearrangement graph for which holds* $\Delta_{\iota }K=\Delta_{du_{1}u_{2}\ldots u_{l}}|K|$ *or* $\Delta_{\iota }|K|=\Delta_{u_{1}u_{2}\ldots u_{l}}|K|$ *for any component subset* K.

## 4. DCJ-INDEL HALVING FOR GENOMES WITH RESOLVED HOMOLOGY

We now have all ingredients to address the GHP for a resolved homology. Similar to Compeau (2013), the following can be shown as in Bohnenkämper (2023b, section A.2).

**Proposition 2.** *The restricted DCJ-Indel halving distance for a genome* G *with circular singletons* $C_{1},..,C_{l}$ *under resolved homology* $\left(\equiv^{⋆}\right)$ *is*

(5) $$\bar{h_{DCJ}^{id}}\left(G,\equiv^{⋆}\right)=\bar{h_{DCJ}^{id}}\left(G\prime ,\equiv^{⋆}\right)+l$$

*where* G′ *contains the same chromosomes as* G, *except* $C_{1},..,C_{l}$.

Circular singletons can therefore be dealt with in preprocessing and we need not consider them from now on. We start by establishing a lower bound for the problem without circular singletons.

**Proposition 3.** *For a genome* G *with a resolved homology* $\left(\equiv^{⋆}\right)$ *containing no circular singletons holds*

(6) $$\bar{h_{DCJ}^{id}}\left(G,\equiv^{⋆}\right)\geq n-c_{\circ }+\left\lceil \frac{p_{g|g}+\max\left(p_{G\circ g},p_{G|g}\right)-p_{G|G}}{2}\right\rceil ,$$

*where* $n=|\left\{\left[m\right]|m\in M_{G},|\left[m\right]|=2\right\}|$.

Recall that $p_{g|g}$ is the number of odd pontoons, that is, odd paths ending in two lava vertices, $p_{G\circ g}$ and $p_{G|g}$ are the numbers of even and odd piers, respectively, which end in a lava vertex as well as a telomere and $p_{G|G}$ is the number of odd viaducts, which end in two telomeres. For an example, see Figure 5. To more easily address individual terms, we use the following shorthands,

(7) $$H:=n-c_{\circ }+Q:=n-c_{\circ }+\left\lceil \frac{q}{2}\right\rceil$$

(8) $$:=n-c_{\circ }+\left\lceil \frac{p_{g|g}+\max\left(p_{G\circ g},p_{G|g}\right)-p_{G|G}}{2}\right\rceil .$$

We start our proof of Proposition 3 by showing the following:

**Proposition 4.** *For a genome* G *with a resolved homology* $\left(\equiv^{⋆}\right)$ *containing no circular singletons holds that* G *is SD iff* $n-c_{\circ }+Q=0$.

*Proof.* If there are no singular markers, *H* reduces to

(9) $$n-c_{\circ }+\left\lceil -\frac{p_{G|G}}{2}\right\rceil =n-\left(c_{\circ }+\left\lfloor \frac{p_{G|G}}{2}\right\rfloor \right),$$

which is the DCJ halving formula by Mixtacki (2008) and, therefore, has already been shown to be 0 if and only if G is SD. If there are singular markers, obviously G cannot be SD, so the forward direction is trivial. For the backward direction, notice that every extremity of a nonsingular marker needs to either contribute to a 2-cycle or 1-path to reduce $n-\left(c_{\circ }+\left\lfloor \frac{p_{G|G}}{2}\right\rfloor \right)$ to zero. Therefore, every adjacency and telomere containing an extremity of a nonsingular marker must have an equivalent. Thus, extremities without an equivalent can occur only as part of singleton chromosomes. Since our premise is that the genome contains no circular singletons, only linear singletons can remain. These, however, contain even piers $P_{G\circ g}$ at their ends, thus $\max\left(p_{G\circ g},p_{G|g}\right)>0$ and with that H>0, a contradiction. Therefore, G must be SD.

We observe that DCJ operations can change *H* by at most 1.

**Proposition 5.** *For any DCJ operation d holds* $\Delta_{d}\left(n-c_{\circ }+Q\right)\geq -1.$

*Proof.* DCJ operations do not affect *n*. As we have seen in Observation 3, if $c_{\circ }$ changes, none of the terms in $Q=\left\lceil \frac{p_{g|g}+\max\left(p_{G\circ g},p_{G|g}\right)-p_{G|G}}{2}\right\rceil$ can change at the same time. Since $\Delta_{d}c_{\circ }\geq -1$ for a DCJ operation *d* (see Corollary 2), we only need to concern ourselves with *Q*. To resolve the maximizations in the formula, we observe that the maximum of two elements changes at most as much as one of the two elements.

**Observation 10.** *For any operation o holds*

(10) Δo max(x,y)≥ΔoxorΔo max(x,y)≥Δoy.

Together with Corollary 1, we are able to derive

**Corollary 3.** *For a given DCJ operation d*, *there is* $x\in \left\{p_{G\circ g},p_{G|g}\right\}$ *with*

(11) $$\Delta_{d}\left(p_{g|g}+\max\left(p_{G\circ g},p_{G|g}\right)\right)\geq \Delta_{d}\left(p_{g|g}+x\right)\geq^{Cor.1}-2.$$

We see that the only way the numerator $q=p_{g|g}+\max\left(p_{G\circ g},p_{G|g}\right)-p_{G|G}$ could be reduced by more than two is if $p_{G|G}$ is increased and another term is decreased at the same time. Because of Observation 5, we know that this cannot be $p_{g|g}$. Since any DCJ operation with a lava vertex in one of its resultants creates at most one viaduct (see Observation 4), the only operations that could decrease *q* by more than 2 are of the form 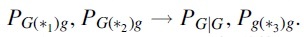 From Observation 6, we know that $\left({*}_{3}\right)=\left({*}_{1}\right)+\left({*}_{2}\right)+|$ and thus, either the sources are of different parity, that is, 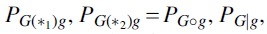 from which follows $\Delta \max\left(p_{G\circ g},p_{G|g}\right)\geq -1$ or one resultant is an odd pontoon, that is, 
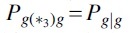
 and therefore $\Delta p_{g|g}=1$. In both cases, we have Δq≥−2 and, therefore, ΔH≥1. Thus, this concludes our proof of Proposition 5. □

We are left to examine the effect of Indel operations. Regarding Observation 7, we see that a uni-Indel either concatenates two piers or pontoons, thereby possibly creating a viaduct or a cycle from an even pontoon. From this follows that a uni-Indel *u* has either $\Delta_{u}c_{\circ }\leq 1$ and $\Delta_{u}Q=0$ or $\Delta_{u}c_{\circ }=0$ and $\Delta_{u}Q\geq -1$. Since none of the component subsets in *H* contains (even) pontoons of length 0, we conclude using Observation 8:

**Observation 11.** *For any deletion* δ *holds* $\Delta_{\delta }H\geq -1.$

Conceptualizing insertions as an insertion of a circular chromosome followed by a DCJ (see Observation 9), we see that the insertion of a circular chromosome with *l* markers increases *n* by *l*. On the other hand, the *l* uni-Indels creating its adjacencies decrease $-c_{\circ }+Q$ by at most *l*. The final DCJ has ΔH≥−1. We thus find:

**Observation 12.** *For any insertion* ι *holds*

(12) $$\Delta_{\iota }H=\Delta_{\iota }n+\Delta_{\iota }\left(-c_{\circ }+Q\right)\geq l-l-1=-1.$$

Altogether, from Observations 11 and 12, and Propositions 4 and 5 follows Proposition 3.


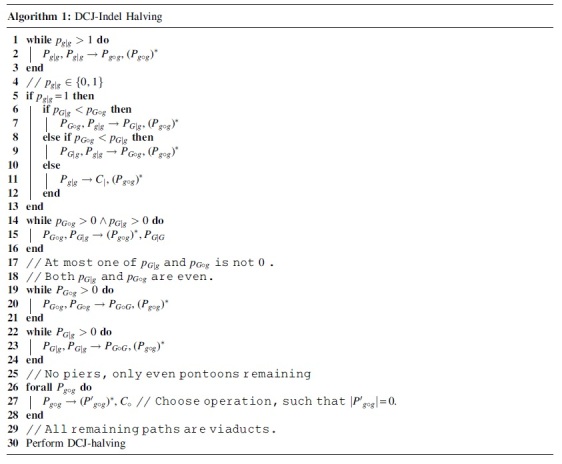


We give a sorting algorithm that attempts to achieve the lower bound given in Proposition 3 in Algorithm 1. Every step in the algorithm is conceived as a DCJ operation X,Y→W,Z transforming X,Y into W,Z. This is not always possible without creating a circular singleton, which can only arise if an even pontoon $Z=P_{g\circ g}$ of length 0 is created. In these cases, we have written the operation as 
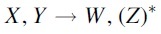
 instead. If creating *Z* would generate a circular singleton, we simply replace the operation by the deletion X,Y→W. This is possible because if *Z* would be part of a circular singleton, it means that there is a chromosome segment connecting *X* and *Y*.

We now regard the assertions written as comments. Most assertions in the algorithm simply follow from the preceding while-conditions. Only the assertion in Line 18 might need some clarification. Each linear chromosome of a genome ends in two telomeres, each of which is associated with exactly one pier and vice versa. Therefore, the number of piers is always even. Since there is only one type of pier in the graph at that point (see Line 17), the number of that type must be even.

Our algorithm reduces *H* by 1 in almost every step by either having Δq=−2 or $-\Delta c_{\circ }=-1$ (Table 1). Note here that the number of piers being even implies that the difference between $p_{G\circ g}$ and $p_{G|g}$ is also even, guaranteeing that $\Delta \max\left(p_{G\circ g},p_{G|g}\right)=-1$ in steps 7 and 9.

**Table 1.** Changes to Terms of *H* in Algorithm 1

Step	$-\Delta c_{\circ }$	$\Delta p_{g|g}$	$\Delta max\left(p_{Gog},p_{G|g}\right)$	$\Delta p_{G|G}$
2	0	−2	0	0
7	0	−1	−1	0
9	0	−1	−1	0
11	0	−1	0	0
15	0	0	−1	1
20	0	0	−2	0
23	0	0	−2	0
27	1	0	0	0

The only step that does not guarantee ΔH=−1 is Step 11, so the algorithm only reaches a modified bound. However, we will show that this new bound is correct.

**Theorem 1.** *For the Maximum Matching halving of a genome* G *with resolved homology* $\left(\equiv^{⋆}\right)$ *without circular singletons holds*

(13) $$\bar{h_{DCJ}^{id}}\left(G,\equiv^{⋆}\right)=n-c_{\circ }+\left\lceil \frac{p_{g|g}+\max\left(p_{G\circ g},p_{G|g}\right)-p_{G|G}+\delta }{2}\right\rceil$$

*where* δ=1 *if* $p_{g|g}$ *is odd and* $p_{G|g}=p_{G\circ g}$, δ=0 *otherwise*.

*Proof.* Algorithm 1 shows that Theorem 1 is an upper bound on the halving distance. What remains to be seen is whether it is a lower bound as well.

We write $H\prime :=n-c_{\circ }+\left\lceil \frac{q+\delta }{2}\right\rceil$. Since δ is non-negative and 0 if the genome is SD, Proposition 4 holds for both *H* and H′. Because of Proposition 3, the only way H′ could be reduced by more than 1 is if Δδ=−1. We discuss DCJ operations first. If $\Delta \max\left(p_{G\circ g},p_{G|g}\right)\geq 1$, after cancellation the terms remaining are $\Delta q\geq \Delta \left(p_{g|g}-p_{G|G}\right)\geq -2$ (see Observations 1 and 5). Therefore, if ΔH′≤−2, we know that the maximization term $\Delta \max\left(p_{G\circ g},p_{G|g}\right)$ is not positive. We now distinguish two cases, in which Δδ=−1.

(I) For the first, let us presume that after the operation, $p_{G\circ g}\neq p_{G|g}$. In conjunction with the fact that a DCJ operation has at most two sources, this means that either $\Delta p_{G\circ g}<0$ or $\Delta p_{G|g}<0$, but not both. Therefore, $\Delta \max\left(p_{G\circ g},p_{G|g}\right)=0$. Plugging this and δ=−1 in the formula, we arrive at $\Delta q=\Delta p_{g|g}-\Delta p_{G|G}-1$. Using Observation 5, we then know that $\Delta q\geq \min\left(\Delta p_{g|g}-1,-\Delta p_{G|G}-1\right)$. As at least one of the sources of the operation must be a pier, we know that $\Delta p_{g|g}\geq -1$ and using Observation 4 that $\Delta \left(-p_{G|G}\right)\geq -1$. Therefore, for this case, Δ(q+δ)≥−2.

(II) Let us now presume that after the operation still $p_{G\circ g}=p_{G|g}$. From Δδ=−1 then follows that $\Delta p_{g|g}\in \left\{-1,1\right\}$.

1. Let $\Delta p_{g|g}=-1$. It then follows that $\Delta \max\left(p_{G\circ g},p_{G|g}\right)\geq 0$, because one source is already “blocked” by the odd pontoon and we would need two piers as sources to reduce the term. Since $\Delta p_{G|G}\leq 0$ (see Observation 5), we have Δ(q+δ)≥−1+0−0−1=−2 in this case.

2. For $\Delta p_{g|g}=1$, we then have $\Delta \left(q+\delta \right)=\Delta \left(\max\left(p_{G\circ g},p_{G|g}\right)-p_{G|G}\right)$. Because of $p_{G\circ g}=p_{G|g}$, we have $\Delta \max\left(p_{G\circ g},p_{G|g}\right)\geq -1$. Using Observation 4, we know that $\Delta \left(\max\left(p_{G\circ g},p_{G|g}\right)-p_{G|G}\right)\geq -2$.

We thus see that in all cases for DCJ operations holds Δ(q+δ)≥−2.

For uni-Indels, we see using Observation 7 that to reduce δ by 1, they can either fuse two piers of the same parity, fuse a pier with a pontoon or form an odd cycle from an odd pontoon. All of these have $\Delta \max\left(p_{G\circ g},p_{G|g}\right)\geq 0$, $\Delta p_{G|G}=0$, $\Delta c_{\circ }=0$, and $\Delta p_{g|g}\geq -1$. Thus, for uni-Indels ΔH′≥−1. Using Observations 8 and 9, we can conclude that Indels also have ΔH′≥−1. Thus, H′ is an upper as well as lower bound for the restricted halving distance for a resolved homology.

Since H′ can be calculated in polynomial time by building the supernatural diagram and traversing the graph identifying the present component types, we obtain:

**Theorem 2.** *Calculating the restricted DCJ-Indel halving distance* $\bar{h_{DCJ}^{id}}\left(G^{\equiv^{⋆}}\right)$ *for a resolved homology* $\left(\equiv^{⋆}\right)$ *can be achieved in polynomial time.*

## 5. GENERALIZATION TO NATURAL GENOMES

We will now generalize what we have seen in Section 4 to arbitrary homologies, that is, *natural genomes*. Since the GHP under DCJ-Indel and Maximum Matching is NP-hard for this case, we give an ILP solution for the problem. Because of the fairly simple distance formula derived in Section 4, our ILP works without computing a capping, that is, the circularization of linear chromosomes. This is in contrast to similar solutions of other problems (e.g., Shao et al., 2015 or Bohnenkämper et al., 2021). The advantage is that the size of our ILP is linear with respect to the number of linear chromosomes, while solutions relying on capping grow quadratically in the number of linear chromosomes, which leads to a dramatic increase in the search space (Rubert and Braga, 2022).

We give the constraints of the ILP, named *hang* for halving natural genomes in Algorithm 2 and its domains in Table 2.

**Table 2.** Domains for Algorithm 2

Binary	General
$x_{e}\forall e\in E_{\xi }\cup E_{\gamma }$	$0\leq y_{v}\leq ix\left(v\right)\forall v\in V\cup V_{⊘}$
$d_{m}\forall m\in M_{G}$	$p^{g|g},p^{G\circ g},p^{G|g}\geq 0$
$z_{v}\forall v\in V\cup V_{⊘}$	n,c,s,T≥0
$b_{v}\forall v\in V\cup V_{⊘}$	
$r_{e}^{R}\forall e\in E_{\gamma }\forall R\in \left\{G|G,G\circ g,G|g,g|g,c\circ ,c|\right\}$	
$s_{d}\forall circ.chrom.D$	
δ,NE,O	

To avoid edge-cases, such as paths containing only single vertices, we nonetheless need to make a modification to the supernatural diagram described in Section 2. First, we add an additional vertex $v_{⊘}$ for each telomere *v* and add the adjacency edge $vv_{⊘}$. We call these vertices *pseudo-caps* and write their set as $V_{⊘}$. This procedure ensures that each component contains at least one adjacency edge. Note that in contrast to Shao et al. (2015) and Bohnenkämper et al. (2021), we do not add additional edges connecting all pairs of caps. We thus only have a linear amount of additional edges and vertices.

Like the aforementioned solutions to other problems, our halving solution solves the problem by finding a *matching* (see Problem 3) on the given homology that minimizes the resulting halving distance. We have seen in Section 2 that this is equivalent to finding the minimally scoring consistent decomposition of the corresponding SNG. The term *consistent* in the literature refers to the fact that an extremity edge $\left(m^{t},n^{t}\right)$ is only allowed to be in the decomposition if and only if its *sibling* $\left(m^{h},n^{h}\right)$ is in the decomposition as well.


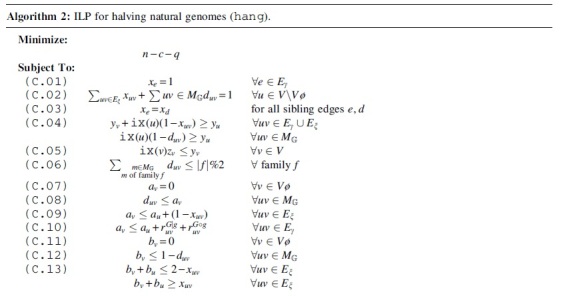
 
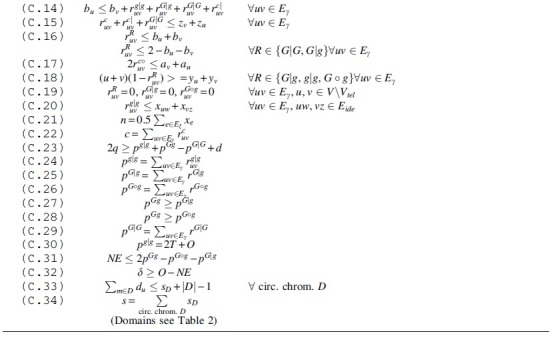


An efficient encoding for consistent decompositions in ILP form was found by Shao et al. (2015) and extended upon for losses in Bohnenkämper et al. (2021). We build upon their solutions and include this general framework as Constraints C.01 to C.05. The constraints compute a consistent decomposition and label each edge *e* with $x_{e}=1$ if and only if *e* is part of the decomposition. We decide whether a vertex *u* is a lava vertex with a binary variable $d_{uv}$ based on the vertex’ marker $uv\in M_{G}$. Exactly one vertex per component without lava vertices (namely *v* with the smallest vertex identifier ix(v)) is labeled with the binary variable $z_{v}=1$. We call this vertex the *reporting vertex*.

We also adapt the way of counting circular singletons from earlier works in Constraints C.33, C.34.

In contrast to Bohnenkämper et al. (2021), we ensure the enforcement of the maximum matching model with a constraint by allowing at most one marker to be deleted if the corresponding family is of odd size (C.06).

The remaining constraints aim to distinguish the different component types (as previously visualized in Fig. 5) from each other. To that end, we define the following binary “report variables” for each adjacency edge *e*: $r_{e}^{X\left(*\right)Y}$ for reporting paths of $P_{X\left(*\right)Y}$ as well as the report variables $r^{c\circ }$ and $r^{c|}$ for reporting even and odd cycles, respectively. The central idea of the ILP is that the corresponding report variable $r_{e}^{Z}$ is set to 1 exactly once per component for an adjacency edge *e* if and only if that component is of type *Z*.

First, we introduce a variable *a_v_* that identifies telomeres (or more precisely, pseudo-caps) by being set to 0 at a pseudo-cap (see C.07) and to 1 at a lava vertex (see C.08). We require this variable to be “passed” through the edges of a component, which is, we require the variable to be the same for two vertices connected by an active edge (see C.09). We only allow a change in this variable within a component if reporting a pier (C.10).

The *a* variable allows us to detect path type based on endpoints, that is, we could now distinguish between viaducts, piers, and pontoons. However, we still need parity to definitely distinguish all types of components. To determine parity, we introduce a variable *b_v_*, which is 0 at the end of a path (see C.11, C.12) and flips upon crossing an extremity edge (see C.13). For adjacency edges, we again require the *b*-variables to be the same, except when reporting an odd component type (C.14).

We now need to ensure that no path of type *Z* could be reported as another type Y≠Z, that is, we need to prevent that *r^Y^* can be set to 1 for a path that is actually type *Z*. Particularly, it is vital that none of the components with a negative or neutral influence on the formula (i.e., $c_{\circ },p^{G|G},c_{|}$) can be reported instead of a component with positive influence (i.e., $p^{g|g},p^{G\circ g},p^{G|g}$) and that components with negative influence are only reported once.

To ensure components with negative influence are only reported once, we only allow them to be reported at a reporting vertex (see C.15). Note that because there is no reporting vertex in components with lava vertices, this constraint also removes the possibility to report a pier or pontoon as a component with negative contribution to H′.

We also ensure that no even component could be reported as an odd component by requiring *b*-variables to be different when reporting odd components (see C.16). Note that odd components can already not be reported as even ones as they require the *b*-variable to flip along at least one adjacency edge in the component.

To avoid paths being reported as cycles, we require there to be no telomeres when reporting cycles (see C.17). To avoid report variables being used to alter parity or *a*-variables in components without lava vertices, we require that no *z* variables are set by setting the *y* variables of the component to 0 (see C.18).

Finally, to canonize where components will be reported, we require viaducts and pontoons be reported at the telomere end (see C.19) and restrict the reporting of pontoons at the lava vertex (see C.20). This requires pseudo-caps to have lower indices than regular vertices ($ix\left(v\right)<ix\left(u\right)\forall v\in V_{⊘}\forall u\in V$), but this is easy to accomplish in preprocessing.

Constraints C.21 to C.29 then summarize the reports to the terms found in the formula.

To set the variable δ detecting the edge case of Theorem 1, we determine whether $p^{g|g}$ is odd in Constraint C.30 and store the result in *O*. Furthermore, we set variable *NE* to 0 if we have the same number of odd and even piers (see C.31). Finally, C.32 determines whether δ must be set.

Note here that the solver can also find an alternative solution where it can increase $p^{g|g}$ or $p^{Gg}$ by 1 and set δ=0. Thus, a solution might actually fulfill the edge case in Theorem 1 while still having δ=0 in the ILP, but instead increasing *q* in another variable. However, since this has the same effect on the formula, we did not introduce more constraints to prevent this behavior.

The ILP has a linear number of constraints and variables with respect to the size of the graph with the vast majority of variables being binary variables.

## 6. EVALUATION

We implemented the ILP introduced in Section 5. The program as well as the evaluation pipeline can be found here: https://gitlab.ub.uni-bielefeld.de/gi/hang.

### 6.1. Simulated data

To evaluate the ILP's performance on genomes with varying characteristics, we extended the simulation program introduced in Bohnenkämper (2023b) to handle simulating arbitrary trees as well as WGDs. For all of our simulations, we used the same linear tree topology, given in Newick format here: (((G)D)S)R;. Since the root *R* by default contains no duplicate markers, the first branch from *R* to *S* serves to mainly introduce duplicates through duplications. We then set the simulation to perform a single WGD on branch *S* to *D*. Lastly, a number of rearrangements, deletions, insertions, and duplications are performed from *D* to *G*. *G* is then used as the input for generating the ILP.

We always set the number of operations to be performed branches *R* to *S* and *D* to *G* to be the same, but set the deletion rate to 0 on branch *R* to *S* as deletions would not have any impact on the scenario before the WGD.

Our default simulation parameters were 5000 markers on one linear chromosome for *R*, 2500 operations performed from *D* to *G* with operations performed according to the following rates relative to the number of DCJs: An insertion rate of 0.2, a duplication rate of 0.3, and a post-WGD deletion rate of 1.0. The length *l* of each indel and duplication is drawn from a Zipf distribution with probability density function $p\left(l\right)=\frac{l^{-a}}{\zeta \left(a\right)}$, where ζ is the Riemann Zeta function and *a* is a shape parameter with a>1. We set the shape parameter *a* for duplications and Indels to 6 and 4, respectively. Whenever we deviated from these parameters in the following experiments, we specify how in the corresponding paragraph.

To obtain ILP solutions, we used gurobi-10.0 running on a single thread on an AMD EPYC CPU on a virtual machine with a set time limit of 1 hour (3600 seconds).

**FIG. 10.**
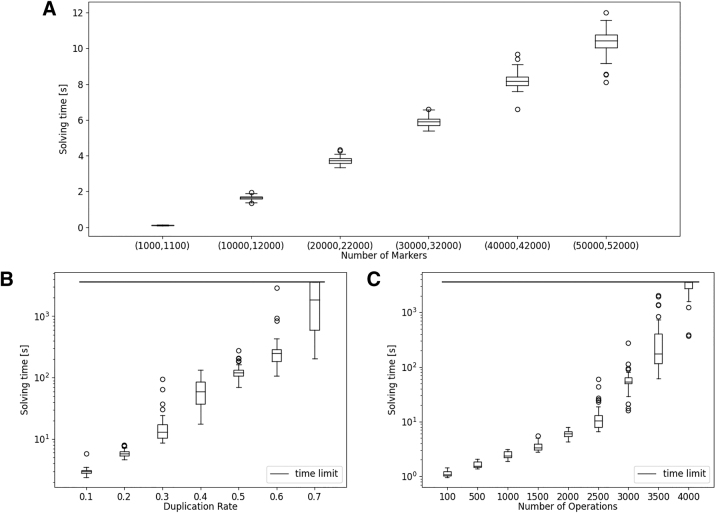
ILP solving times for: **(A)** genomes with varying numbers of markers; **(B)** genomes simulated with varying duplication rates; **(C)** genomes with varying numbers of operations performed by the simulation. We generated 50 samples per bucket in each experiment.

For our first experiment, we examined the influence of the genome size on the ILP performance. To that end, we simulated root genomes (*R*) of sizes 1000 to 25,000 leading to final genomes *G* of sizes 2000 to 50,000. Since the number of rearrangements in real typically correlates strongly with genome size, we set the number of rearrangements performed by the simulation to 10% of the root genome size. The resulting solving times (see Fig. 10A) indicate that genome size alone at least in the range tested is not a major factor. While the solving times do rise, they do so only linearly and do not go beyond a few seconds.

Arguably the strongest influence on the size of the solution space is the number of extremity edges in the graph, that is, the number of ambiguous markers in the genome. We, therefore, performed an experiment varying the duplication rate in the simulation from 0.1 to 0.7 in steps of 0.1. The results are shown in Figure 10B. We observed an exponential increase in both the solving times and their variance with 20 samples of the 0.7 tier not being solved within 1 hour (3600 seconds).

Another factor we can expect to influence runtimes is the complexity of the resulting supernatural diagram, that is, the length and nesting of the paths and cycles of its decompositions. Since this complexity rises with more rearrangements applied on the branch after the WGD, we performed an experiment gradually increasing the total number of rearrangements, Indels, and duplications performed from 100 to 4000. The runtime results are found in Figure 10C. We observed a similar, if not more drastic increase in solving times in this experiment, with even 33 samples of the last tier exceeding the time limit.

Compared with related ILPs, such as that in Bohnenkämper et al. (2021), hang does not perform as well as expected. While its size scales linearly with the size of the graph, we hypothesize that the SNG of a natural genome is on average more complex than the graph data structure used for distance comparisons of a pair of natural genomes. For example, the graph used in Bohnenkämper et al. (2021) is bipartite with respect to extremity edges, possibly leading to less nested structures from ambiguous markers.

**FIG. 11.**
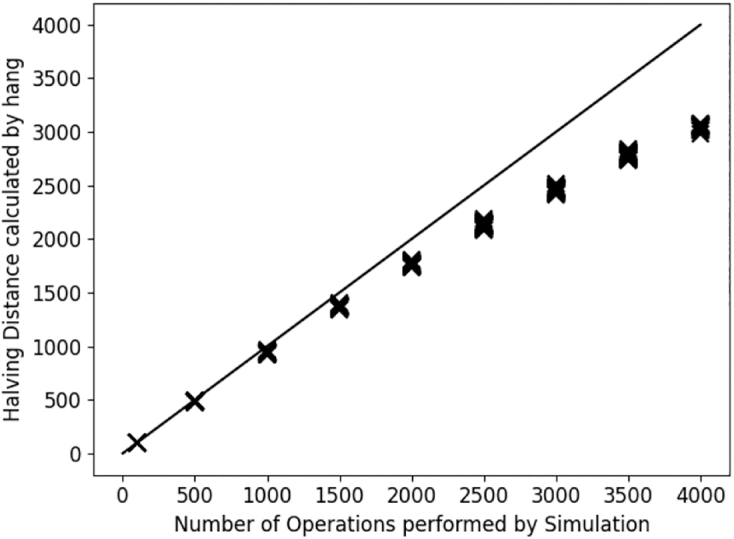
Number of simulated operations compared with the halving distance as calculated by hang. The line shows the identity function.

To test the accuracy of the halving distance calculated by hang, we retained the optimal solutions of the last experiment and compared the calculated halving distance to the simulated distance. This evaluation is shown in Figure 11. We see that for low distances hang calculates results very close to the simulated number of operations before tapering off to be consistently below the simulated distance. Notably, the variance of the calculated distances is small compared with the deviation from the simulated distance. This effect is reminiscent of the behavior of classic sequence edit distances, in which back mutations and similar effects shorten the minimal edit distance while the actual evolutionary distance grows. Given that this effect can be corrected for in classic edit distances, we conjecture that this could be done for halving distances as well if one finds a sufficiently powerful statistical modeling of DCJ, Indel, and duplication operations. However, one possible obstacle for this statistical correction is likely the dominance of losses after a WGD, which might more strongly obscure rearrangements in real data.

Overall, our simulated experiments show that the performance of hang is reasonable as long as not too many rearrangement events occurred after the WGD, although not as high as one would expect from related ILPs. Moreover, the calculated halving distances can be meaningfully interpreted, provided the effect of marker losses can be bounded.

### 6.2. Examining the yeast WGD

To evaluate the efficacy of our method in practice, we analyzed a real example of a WGD.

We, therefore, downloaded the GenBank files of three yeast genomes known to have undergone a WGD before diverging (Scannell et al., 2006), namely those of *Saccharomyces cerevisiae*, *N*aumovozyma *castellii*, and *N*akaseomyces *glabratus*. We proceeded to extract the longest coding sequences using a script of the software package FFGC (Doerr et al., 2018) and performed an all-vs-all BLAST (Altschul et al., 1990) comparison. We retained hits with an *e*-value of 0.01 or lower and created families according to these hits. For maximum sensitivity, we defined the families in a transitive manner, that is, we would assign markers a,b,c the same family even if only (a,b) and (b,c) were significant hits, but not (a,c).

We let our software create the ILPs and calculated solutions using gurobi-10.0 with 10 threads and a time limit of 9 hours on an Intel Xeon E7540 CPU.

The results are shown in Table 3. For two out of three runs, gurobi found an exact result within the time limit and an approximate result with a gap of 3.87% for *S. cerevisiae*. While the halving distances calculated (Column obj.) are broadly in agreement for *N. castellii* and *S. cerevisiae*, the halving distance of *N. glabratus* is significantly lower than the others. One possible explanation for this fact reveals an important limitation of our method.

**Table 3.** Genome Assemblies and Basic Statistics for the Three Taxa Used in Our Experiments

Taxon	Assembly	#Markers	#Non-sing.	#Amb. f.	Max. fs.	Obj.	Gap (%)
*Naumovozyma castellii*	GCA_000237345.1	5590	469	28	15	356	0.00
*Saccharomyces cerevisiae*	GCA_000146045.2	5943	635	42	48	413	3.87
*N*akaseomyces *glabratus*	GCA_000002545.2	5201	237	20	18	117	0.00

#amb. f., the number of ambiguous families, #markers, the number of coding sequences used as markers; #non-sing., the number of nonsingular markers; Assembly, Accession.version; gap, the gap reported by gurobi upon reaching the time limit; max. fs., the maximum family size; obj., the halving distance calculated as the objective by gurobi; Taxon, the taxon name.

Comparing the numbers of nonsingular markers, we can see that while all genomes consist mainly of singular markers, *N. glabratus* has a particularly low number of nonsingular markers. This is relevant, because—as in many rearrangement studies—the number of rearrangements detectable by the DCJ-Indel model in the halving problem is limited by the number of markers in the resulting matching. This can be seen by noting that the positive terms in the formula that do not depend on the number of chromosomes, namely *n* and $p_{g|g}$, rise at most linearly with the number of markers in the matching. Therefore, a lack of nonsingular markers can easily yield underestimated results for the halving distance like we observed here.

One possible solution to this problem might be an affine cost model that scores both the number of Indel operations as well as the length of the segments deleted.

## 7. CONCLUSION

After presenting some general statements about a generalization of simple SNGs, we were able to derive a compact formula for the halving distance under the DCJ-Indel model that can be computed in polynomial time for genomes with resolved homology. We note that the problem is NP-hard on arbitrary homologies. However, due to its compactness, the formula is generalizable into an ILP for natural genomes.

The ILP performs reasonably well on simulated genomes up to a few 10,000 markers, provided the genomes contain not too many duplicates and are not strongly rearranged. For real genomes the approach looks promising at least in terms of performance, although care needs to be taken in interpreting its results, particularly if the genome in question lacks sufficient nonsingular markers.
